# Training in critical care echocardiography

**DOI:** 10.1186/2110-5820-1-36

**Published:** 2011-08-30

**Authors:** Paul H Mayo

**Affiliations:** 1 Long Island Jewish Medical Center, 270-05 76th Avenue, New Hyde Park, New York 11040, USA

## Abstract

Echocardiography is useful for the diagnosis and management of hemodynamic failure in the intensive care unit so that competence in some elements of echocardiography is a core skill of the critical care specialist. An important issue is how to provide training to intensivists so that they are competent in the field. This article will review issues related to training in critical care echocardiography.

## Introduction

Echocardiography has unparalleled utility in the intensive care unit (ICU). It allows the intensivist to assess rapidly the anatomy and function of the heart in patients with hemodynamic failure. This allows the clinician to make immediate visual diagnosis and to guide the ongoing management of the case. Its ease of use, bedside utility, and quality of information make cardiac ultrasonography a key skill for the frontline intensivist. Given the importance of echocardiography in the ICU, an important issue is how to provide training to intensivists so that they are competent in the field. This article will review the issues related to training in critical care echocardiography (CCE).

This article is of interest for two groups of intensivists. For the intensivist who does not have training but who seeks to develop competence in CCE, this article will be helpful in providing a guide to the training process. For the intensivist who already has training but who has the additional responsibility to train other clinicians to become competent in CCE, this article will be helpful as a framework for developing teaching process.

In the United States, there are approximately 6,000 intensivists at the attending level who need training in CCE. These are frontline attendings who seek to use CCE as a primary bedside imaging modality and who work on a full-time basis in the ICU. Some of these are faculty intensivists who have responsibility for critical care fellows who need training in CCE. In Europe, it is difficult to estimate the number of intensivists who need training, because each country has individual patterns of unit staffing. It is likely that the numbers are similar in magnitude to the United States. In Australia, there are approximately 400 fulltime intensivists who need training in CCE. It is not possible to estimate the numbers in other countries of the Asia Pacific region, Asia, Africa, or South America. China and India alone may add many thousands more to the count in the coming years. The challenge to the individual intensivist is how to achieve competence in the CCE, whereas the challenge at the system level is how to design training methods that can efficiently and effectively train many thousands of intensivists [1-3].

A key element to the design of a process of training is to define explicitly the goals of training. Competence is the goal of training. To design a training program, there must therefore be a specific definition of competence in CCE. In 2008, a working group comprised of representatives from France and the United States coauthored a statement that defined competence in critical care ultrasonography [4]. The document, American College of Chest Physicians/La Société de Réanimation de Langue Française Statement on Competence in Critical Care Ultrasonography, includes explicit discussion of CCE. CCE is divided into two levels of competence: basic level CCE and advanced level CCE. Basic level CCE emphasizes a goal-directed examination of the heart with a limited number of standard views (e.g., parasternal long and short axis, apical four chamber, subcostal, and inferior vena cava), which are used to categorize shock state and to guide management of the patient with hemodynamic failure. Color Doppler is used as a method to screen for severe valvular dysfunction, but basic CCE includes no other component of the Doppler examination. Competence in basic level CCE is a key element of competence in critical care ultrasonography.

Advanced-level CCE requires that the clinician achieves competence in aspects of echocardiography that are part of standard cardiology-type echocardiography, in addition to achieving competence in elements of echocardiography that are particular to critical care medicine. Competence in advanced-level CCE requires a long course of study similar in complexity to the cardiologist who is trained in echocardiography. It includes training in transesophageal echocardiography (TEE). Competence in advanced level CCE is not a key element of competence in critical care ultrasonography. The majority of intensivists do not have the time, the interest, or the need for advanced level of training. It is not clear what proportion of intensivists should acquire advanced level. One approach in a large ICU that is staffed by a full-time intensivist team is for several team members to have advanced CCE training, whereas all other team members have basic CCE skill. This allows ready access to advanced CCE capability, should the clinician with basic level CCE skill need backup.

The importance of the Statement on Competence is that it clearly defines competence in both types of CCE. It therefore has utility for both the trainee and the trainer, because it provides a road map for training. It has been adopted as the foundation document for a multinational consensus statement on training in critical care ultrasonography.

Following the development of the Statement on Competence, a working group met in 2009 to develop guidelines for training in critical care ultrasonography. Representatives from major critical care organizations of Europe, North America, America, the Middle East, and the Asia-Pacific region met under the aegis of the European Society of Intensive Care Medicine. The resulting document, International Expert Statement on Training Standards for Critical Care Ultrasonography, is based on consensus of the group and addresses the question of how to train to become competent in both basic and advanced CCE [5].

For training in basic CCE, the working group decided that the theoretical component of training should include a minimum of 10 hours of course work, combining lecture, didactic cases, and image interpretation. The learning may utilize a blend of lecture format and internet-based material. For training in image acquisition, a minimum of 30 fully supervised studies is suggested as a reasonable target. Initially, training scans may be performed on normal subjects. A key part of training includes a component of bedside scanning of patients in the ICU under the supervision of a local expert who is competent in basic CCE. For training in image interpretation, the trainees should be exposed to a comprehensive collection of abnormal images, because it is not expected that they will see all important pathology during their image acquisition training. The trainee should keep a logbook of scanning activity and make formal readings of their scans under supervision of their trainer. Training in TEE is an optional component of basic CCE.

Training in advanced CCE requires a minimum of 40 hours of course work using the same techniques described for basic CCE. Image acquisition training requires a suggested minimum of 150 transthoracic (TTE) and 50 TEE studies performed under the direct supervision of a local expert. In other respects, training methods are similar as with basic CCE, using blended techniques for cognitive training, image interpretation from a comprehensive image collection, and initial training with normal subjects followed by extensive bedside scanning under direct supervision of a local expert.

The working group decided that a formal certification process for basic CCE was not required but that a certification process was required to ensure competence in advanced CCE given the complexity of the field and the need for recognition of high skill level by colleagues and administrative entities.

The Statement on Competence is very specific. The Statement on Training is less so. For example, the requirement for number of studies needed for training is a suggested target, because the group felt that there was insufficient evidence to make a more definitive recommendation. The document establishes a broad standard to allow maximum flexibility in design of training programs in a wide variety of medical cultures. However, one unequivocal statement is as follows: basic level critical care echocardiography and general critical care ultrasound should be a required part of the training of every ICU physician [5].

When undertaking training in CCE, the trainee may ask whether intensivists can actually perform the procedure. The emphatic answer must be in the affirmative. There is nothing intrinsic to cardiology training that limits echocardiography to the cardiologist. It is simply another imaging technique that can be learned by any interested clinician; but is there evidence that supports this contention? Many groups have demonstrated that noncardiologists can be trained to perform components of the basic CCE examination with reliable results [6-15]. The American Society of Echocardiography have issued a recent position paper that supports the use of limited echocardiography by emergency medicine physicians--a group that has close parallel to the critical care specialist [16]. In reference specifically to intensivists, Manasia et al. reported on the utility and accuracy of goal-directed TTE performed by intensivists with positive results [17]. Vignon et al. observed that critical care residents could master basic CCE with results similar to expert level echocardiographers [18]. It is clear that intensivists can become competent in basic CCE.

Regarding advanced CCE, there are well-defined training tracks that produce intensivists who are clearly competent at an advanced level. This reality exists without the need for study.

Although the Statement on Training Standards offers suggestions for target number of studies, there is now evidence that supports these recommendations. Vignon et al. reported that approximately 33 TTE are adequate for training in basic CCE for most critical care residents when combined with a 12-hour learning program blending didactics, interactive clinical cases, and tutored hands on sessions [19]. Charron et al. reported that approximately 30 studies are required for reasonable competence in limited TEE [20]. This study is of particular interest, because it examined the progression of skill acquisition during training and describes a methodical approach to testing for competence in TEE. Benjamin et al. reported that only ten TEE studies were required for intensivists to become competent in screening TEE, but the complexity of the examination was less than that used in the Vieillard-Baron study [21].

Training should result in competence, but this is not assured. Much depends on the motivation of the learner. Poorly structured didactic lectures may not be effective in knowledge transfer. Key to the training process is an interested and effective bedside expert who has the patience to supervise the inexperienced trainee. Numerical goals may foster the attitude that competence may be achieved by performing a large number of low-quality scans as rapidly as possible. Instructors will note a wide range of intrinsic talent in the learners at a course. Some lack the eye-hand coordination required for effective transducer manipulation while others have an intuitive grasp of the subject. Learners progress at their own pace. Defining the training process and sending the learner through a well-defined training process does not guarantee competence.

This raises the issue of certification. The argument in favor of a formal certification process for CCE is that it may ensure that the participant has reached some predefined skill level. It defines an important minimum standard. Presumably, this would yield better results at the bedside. There are several arguments against developing a formal certification process for basic CCE. Assuming that basic CCE is a key skill for all intensivists, a large number of intensivists would need to go through the certification process. This presents logistical problems that are compounded by the complexities related to transnational differences of medical training practice and testing methods. It is likely that different national societies would want to design their own certification process. For a certification process to by truly meaningful, it must be designed by an agency that is completely independent of the training system to avoid conflict of interest.

In the United States, the authority responsible for developing the highest standard of certification, the American Board of Internal Medicine, has not been interested in developing a certification process for a relatively small aspect of critical care medicine. The time and expense required to develop a high-grade certification process are considerable and do not warrant resource allocation for a skill that is of importance to only small group of clinicians. This is likely to be the case in other countries as well. Basic CCE is only a small part of the large skill set required for the practice of critical care medicine. If certification in basic CCE is a requirement to demonstrate competence, why not require certification in other aspects of critical care medicine that are clearly of much higher risk and complexity, such as airway management, vascular access, or ventilator management? This argument favors the position that basic CCE should be bundled into other important aspects of critical care practice that do not require individual certification.

By common consensus, training in advanced CCE requires formal demonstration of competence, if possible with certification by an agency that is independent of the training system. This ideal has been achieved in France, where the critical care community has developed a structured pathway for training in advanced CCE that leads to certification. During the first year, the fellow trains in echocardiography alongside cardiology fellows and is required to perform at least 120 TTE studies. During the second year, they continue their training under the direct supervision of an expert-level critical care echocardiographer. They are required to perform an additional 120 studies, of which 50 must be TEE. They are mandated to attend a standard set of didactic courses. On completion of these requirements, they must pass a high stakes board-type examination that has a significant failure rate. During the first 2 years of operation of this program, 200 intensivists achieved certification in advanced CCE, and 39% of fellowship programs are certified to provide the training.

The French certification process, which was developed with the support of the national cardiology and anesthesiology societies, is clearly a model for others to follow [1]. A similar training track is available for attending level intensivists and anaesthesiologists, which requires 40 hours of didactic training and performance of 100 TTE and 50 TEE studies (25 performed and 25 reviewed).

In Australia and New Zealand, the critical care community has developed a two-tier system for training in CCE. Basic level training requires 10 hours of course work and 30 TTE studies with logbook documentation and written report with guidance of a supervisor. No formal examination is required for basic CCE.

The Australian Society of Ultrasound in Medicine (ASUM) has developed a certification process for advanced CCE. The Diploma in Diagnostic Ultrasound (DDU), well established in Australia and New Zealand for the past two decades, catering to radiologists, cardiologists, and obstetricians, has been extended to CCE. The DDU in critical care was offered for the first time in 2010. It consists of two examinations: the first part is a physics examination common to all the different DDU subgroups, and a second examination orientated to the critical care physician. The practical requirements for advanced CCE are similar to those outlined by the International Expert Statement on Training Standards for Critical Care Ultrasonography in terms of number of studies and the need for a logbook and supervisor. Intensivists who successfully complete the training program and the examination obtain a well-established qualification that is recognized throughout both Australia and New Zealand.

In the United States, there is no formal means for the intensivist to achieve high-level certification in advanced CCE. The National Board of Echocardiography (NBE) has established a policy that they will provide certification in echocardiography only to physicians who have completed full fellowship training in cardiology. There is no plan for the board to develop a separate certification track for CCE similar to that in France or Australia/New Zealand. However, the NBE allows any licensed physician to take the echocardiography boards, including intensivists. Curiously, many cardiologists have decided not to take the echocardiography boards. This means that they cannot receive certification in echocardiography by the NBE. As an alternative approach, cardiologists who elect not to take the examination may choose another pathway to demonstrate competence in echocardiography, which is described in a statement developed by the major cardiology societies [22]. Intensivists also may satisfy these requirements, and, if they do, they are competent in echocardiography to equal degree as a cardiologist. The best approach for the intensivist in the United States is to satisfy the requirements of the cardiology statement and also to pass the echocardiography boards. The boards are not required to fulfil requirements for competence. So why take them? The reason is that the intensivist should seek to demonstrate the highest level of capability when presenting themselves as trained in advanced CCE.

Training fellows is less challenging than training attending level intensivists who did not have opportunity to gain experience during their fellowship years. For training fellows, each medical culture will arrive at its own solution for designing an effective training sequence for basic and advanced CCE. The French and Australian/New Zealand system of fellow training is particularly relevant. As to the challenge for clinicians following their fellowship years, it may be instructive to review one approach to the problem of training large numbers of attending level critical care clinicians in basic level CCE

In the United States, the American College of Chest Physicians (ACCP) has developed a program designed for the attending intensivist who seeks training in critical care ultrasonography (thoracic, cardiac, abdominal, and vascular). The total training sequence requires 7 days of course attendance (3-day course followed 4 months later by a 4-day consolidation course), 20 hours of internet based training [23], performance of a 300 image portfolio, and a high stakes board-type examination. The examination includes scripted hands on examination with a human model where the trainee is required to demonstrate skill at image acquisition. The program is designed to give the participant training in all aspects of critical care ultrasonography, including basic CCE. Of the 56 hours of mandatory course attendance, 28 hours are devoted to basic CCE (4 hours didactic lectures, 12 hours of image interpretation training, and 12 hours of hands on training with a faculty trainee ratio of 3:1). The internet-based training has 12 hours that covers basic CCE. The trainee must submit 30 five-view basic echocardiographic studies of acceptable quality for a total of 150 video clips that are reviewed and accepted or rejected by a faculty reviewer. If the trainee passes the examination, they receive a certificate of completion. The ACCP has declined to label this as certification, because they feel that the American Board of Internal Medicine must be involved in the process as an external agency.

## Conclusions

Basic level critical care echocardiography should be a required part of the training of every ICU physician. The Statement on Competence and The Statement on Training Standards serve as useful guides both for the intensivist who seeks training and for faculty intensivists who will be training their colleagues in this important skill.

## Competing interests

The authors declare that they have no competing interests.
